# Belgium’s Healthcare System: The Way Forward to Address the Challenges of the 21st Century: Comment on "Integration or Fragmentation of Health Care? Examining Policies and Politics in a Belgian Case Study"

**DOI:** 10.34172/ijhpm.2022.7070

**Published:** 2022-03-15

**Authors:** Jan De Maeseneer, Anna Galle

**Affiliations:** WHO Collaborating Centre on Family medicine and PHC, Department of Public Health and Primary Care, Ghent University, Ghent, Belgium.

**Keywords:** Integrated Care, Health System Reform, Primary Healthcare, Health Policy

## Abstract

In this paper we have tried, starting from the results of an analysis of the functioning of integrated care in the Belgian Health System by Martens et al, to design a strategy that could contribute to better addressing the challenges of the 21st century in Belgium. We proposed health system changes at the macro-, meso- and micro-level. We focused on health policy development and organization of care, emphasizing the importance of a shift from a hospital-centric towards a primary care based approach. Special attention was paid to the need for institutional reforms, in order to facilitate the further development of interprofessional integrated care, that focuses on the achievement of the life-goals of a person.

## Organization and Integration of Care: The Belgian Context

 In their article: “Integration or Fragmentation of Health Care? Examining Policies and Politics in a Belgian Case Study,”^1^ Martens et al examine the policy process in relation to three integrated care projects of the last decade in Belgium. The authors conclude that “there is a strong stakeholder support for a change towards a more patient-centered system, but that this intention is not consistent with the current provider-driven system and institutional design. Belgium’s political structure is characterized by too much fragmentation and inertia to change, in addition to power imbalances.” These conclusions are relevant when examining the recent performance indicators of the 2021 Belgium Country Health Profile report.^2^ While Belgium is known for a well-developed health sector^3^ some of these indicators show a gloomy picture: potentially avoidable hospital admissions for chronic conditions such as asthma and chronic obstructive pulmonary disease were in Belgium well above the European Union (EU) average in 2019.^2^ Furthermore access to care seems uneven: about 4% of people in the lowest income quintile reported unmet medical needs in 2019, mainly due to costs, compared with 0.2% in the highest.^2^ This gap between the poorest and richest quintiles is the largest among all western EU countries and above the EU average. Moreover the share of health spending paid directly by households (co-payments) is still 18% – a higher share than that in the EU overall (15%).^2^ These findings suggest optimal integration of care to manage chronic conditions is still lacking together with a problem of health equity and inclusiveness.

 These insights are not new, and ask for a fundamental reflection on how a country with a complex structure like Belgium can engage in a sustainable process of change to address the challenges of the 21st century. In 2019, we wrote a policy brief on: “Healthcare in Belgium in 2030: towards a decentralized healthcare system in a solidarity society.”^4^ Shortly after our policy brief, in early 2020, the health system has been overwhelmed by the coronavirus disease 2019 (COVID-19) pandemic. Overall, the COVID-19 has forced healthcare workers in Belgium and beyond more than ever to work more interdisciplinary and adapt to new processes. Furthermore there has been an increased recognition for guaranteeing respectful and person-centered care, especially for older adults.^5^ We hope COVID-19 will bring some sustainable improvements in care processes, serving as a silver lining to the otherwise dark sky of the COVID-19 pandemic. This paper presents some of our suggestions for these improvements, fueled by the 2 years of COVID-19 experience.

## Health System Challenges at the Macro-Level

 Looking at the macro-level, Belgium faces an important problem of complexity. In previous state reforms, the federated entities in Belgium have been designed in “communities” (responsible for the “person-related competencies,” including health and welfare), and “regions” (responsible for the “place-based competencies,” eg, work, infrastructure, economy, etc). This has been leading to very complex situations (with nowadays 9 Ministers in Belgium that have health competencies in the portfolio) and fragmentation. In Brussels for example, there are 3 ‘communities’ active: the Dutch-speaking, the French-speaking and a ‘Joint Communities Commission.’ We believe this approach needs to be revised. Especially the arguments to conceptualize ‘health and welfare’ as ‘place-based competencies’ have become more obvious in light of the COVID-19 pandemic. COVID-19 has demonstrated the importance of a territorial approach to healthcare to contain the spread of the pandemic: home-care happens in the local community, requiring local infrastructure and intersectoral cooperation with housing and work. Recently also the burden of mental health problems has received more attention due to COVID-19: community mental health is an important strategy to tackle psychological problems and the neighborhood is a key element in building social cohesion.

 By opting for the latter approach (‘health and welfare’ as ‘place-based competencies’) the federal state of Belgium could consist of 3 regions where health policies are implemented in order to achieve the health goals approved by the federal parliament: Flanders, Brussels and Wallonia. The federal Minister of Health and the three regional Ministers could then cooperate in the framework of the “Inter-Ministerial Health Conference” and provide the needed ‘vertical integration’ of policies at the federal and regional macro-level. Importantly, there should be an hierarchy and subsidiarity in competencies. Hierarchy means that for the competencies in the federal portfolio the final decision is with the federal Minister. Subsidiarity means that the regions have competencies in the implementation, organization, infrastructure, quality assurance and financing of all activities in health and welfare (prevention, promotion, primary care, secondary care, rehabilitation), except for those topics that are specifically defined in the portfolio of the federal Minister, such as disaster-management, pandemics, food security, air and noise pollution, registration of medication and price and reimbursement of medicines, implementation of international regulations on health professionals, representation of the country health system at international level, availability of highly specialized care for rare and complex conditions. Moreover the federal Minister is responsible for the solidarity collection and distribution of the resources for healthcare. Nowadays the resources for healthcare in Belgium are mainly collected through the labor-tax-financed Social Health Insurance system. One can wonder whether this approach will be sustainable in the future or whether a shift towards a general tax based system will be more appropriate.^6^

 The health budget for the 3 regions, collected through federal level solidarity, should be distributed according to indicators of need (demographic, morbidity, socio-economic, etc). Furthermore the contribution of the regions to the achievement of the health goals should be transparently monitored. In the past, the assignment and financing of the responsibilities to the federal state on the one hand and the federated entities (actually the ‘communities’) on the other hand, has been a major bottleneck, leading to fragmentation of care, inappropriate incentives and loss of efficiency. In our proposal financing of prevention, health promotion, care and cure are located at the same (regional) level. In this manner, economies resulting from appropriate investments in prevention remain in the region and can be used for innovation. The challenge for the forthcoming seventh State Reform (2024?) will be to clearly define the competencies in a coherent way.^7^ Moreover, there will be need for a transition-period, to transform the actual federal payment mechanisms – mostly based on fee-for-service – to more comprehensive global payment systems, that can be used by the regions. For the payment of general practitioners, there is a plan for a shift from primarily fee-for-service to a mixed payment: 60% capitation, 30% fee-for-service and 10% ‘other payments’ (interprofessional cooperation, quality, etc).^8^ Belgium can also take advantage of its almost 40 years of experience with an integrated interprofessional needs-based capitation system for payment of interprofessional primary care teams in “Community Health Centers” and “Medical Homes” with nurses, family physicians, physiotherapists and many other disciplines. Importantly, the assessment of this system, taking care of 0.5 million citizens, indicated that this integrated approach increases accessibility and continuity, enhances prevention and demonstrated better performance (eg, in antibiotic prescription and other quality indicators). Within this system of “Community Health Centers,” integrated care is also sustainably imbedded and does not depend on the voluntary commitment of health professionals, which was described by Martens et al as one of the bottlenecks of the Belgian health system.^1^ These days a future-oriented reflection has started in the National Institute for Health and Disability Insurance to shift from a management based on mono-professional-vertical silo’s (for financing and policy development) towards a more integrated interprofessional and intersectoral transversal approach. Additionally we believe more representatives of citizens/patients need to be included in the decisional processes together with independent scientific advisory boards.

## Health System Challenges at the Meso-Level

 At the meso-level, the regions are responsible for organising the collaboration between the providers and care organizations in health and welfare. In 2019, the Flanders Region decided to territorially organize the meso-level in 60 primary care zones (PCZs), each taking care of 100 000 inhabitants.^9^ This was the result of a participatory process during 10 years with all stakeholders involved. The governance of the PCZ is in the hands of a local “Care Council,” integrating primary healthcare (PHC) services, social services, organizations of patients and informal care givers, and representatives of the local authorities from the cities and villages involved in the PCZ. When the PCZs started their activities in 2020, the first item on the agenda was organizing the primary care response to the pandemic. A “COVID-19 cell” coordinated the actions: early diagnosis of cases by family physicians and timely referral to hospitals, support of chronically ill by nurses both in the community and the heavily affected nursing homes, contact-tracing and source-finding, outreach to vulnerable groups, provision of mental healthcare, and support of quarantine for people living in difficult conditions (eg, poor, homeless, undocumented people).^10^ From 2021 onwards PCZs were asked to organize 1 or 2 Vaccination Centres per PCZ. A unique cooperation between primary care physicians, nurses, pharmacists, social workers and hospital staff, supported by thousands of volunteers made the Vaccination campaign in Flanders a success (Flanders has in 2021 continuously been in the top-5 of the European regions for primo-vaccination and boosters). The crisis of the pandemic enhanced the interprofessional cooperation and linkages between hospitals and PCZs. Moreover family physicians (decentrally) and social insurance organisations (centrally) developed a General Data Protection Regulation (GDPR)-proof system to identify 1.5 million patients at increased risk for COVID-19 (due to co-morbidities), receiving a priority vaccination. Pharmacists played an important role in sensibilization and motivation of patients to become vaccinated. Nurses and mobile teams took care of the vaccination of hard-to-reach groups (homeless, refugees, undocumented people, …). There are indications that the learning during this ‘cooperation under pressure in the pandemic’ also facilitated integrated care for patients with chronic conditions. The PCZs were supported at regional level by VIVEL, the Flemish Institute for Primary Care (https://www.vivel.be/en/) providing webinars, trainings and information materials. The first evaluations of the impact of PCZs are very positive: they are very performant in bringing all primary care actors together and have been strengthening the role of local authorities in the health and welfare system. Currently the way PCZs can be linked functionally to ‘Hospital-networks’ is explored.

## Health System Challenges at the Micro-Level

 Within PCZs, the micro-levelneeds to be refined: a processof care and service delivery putting the person receiving care and support in a central position. Primary care providers in Belgium are more and more shifting the care paradigm from a disease-oriented approach towards a ‘goal-oriented’ approach, focusing on the life-goals of the patient, on what really matters to him/her.^11,12^ Belgium has – except for the community health centres and ‘Medical Homes’ – a limited tradition of interprofessional teams that work with the patients they are accountable for. Nowadays, every patient can choose a set of providers, forming the ‘team.’ This is a rather inefficient way of cooperation, as the interprofessional teams almost never work in the same composition jeopardizing quality integrated care. We suggest to create ‘Primary Care Networks’ at the micro-level, bringing the patients and (health and welfare) professionals of 3 to 5 Family Medicine practices together (minimal 10 000 patients in urban areas, 5000 in rural areas). An important strategy to make this integration happen will be the creation of an “interprofessional integrated goal-oriented electronic health record.” This concept entails that all information of one person is structured based on episodes of care on an electronic platform, filled in by the patient and by the different care providers (GDPR-proof). Central in the design of the electronic health record are the ‘life-goals’ of the person. Every episode of care contributes to the achievement of objectives that enhance the realization of these life-goals. See Figure.

**Figure:**
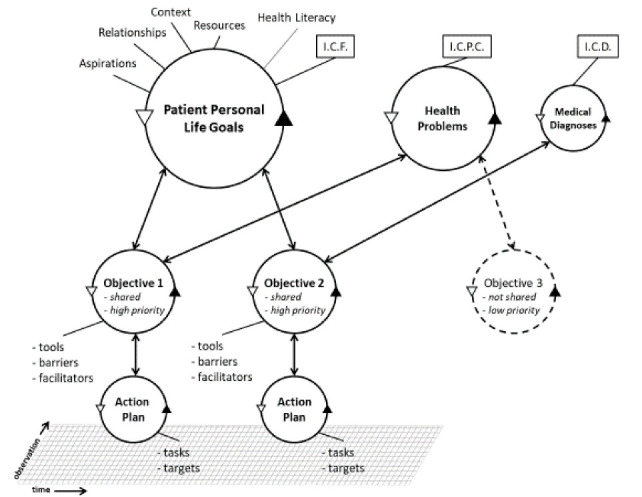
Revised Model of Goal-Oriented Care With The Patient’s Personal Life Goals in Pole Position. Arrows represent flows of information (© 2017 The Author(s). Published by Informa UK Limited, trading as Taylor & Francis Group under the terms of the Creative Commons Attribution License).^13^ Abbreviations: ICD, International Classification of Diseases; ICPC, International Classification of Primary Care; ICF, International Classification of Functioning.

## Conclusion

 In this paper we have tried, starting from the results of an analysis of the functioning of integrated care in the Belgian Health System by Martens et al,^1^ to design a strategy that could contribute to better addressing the challenges of the 21st century. We actually see a dynamic for change with the new federal Minister of Health in Belgium. We proposed health system changes at the macro-, meso- and micro-level and specific recommendations have been made, providing guidance for pilot projects and future research.

 We focused on health policy development and organization of care, because -as in many countries - those changes are needed to shift from a hospital-centric system towards a primary care based system. Nowadays, according to OECD (Organisation for Economic Co-operation and Development)Health Statistics 2018, 22 OECD countries spent on the average 13.6% of their total health expenditures on primary care services, ranging from 18.3% for Australia, 14% for Belgium, to 9.5% for Switzerland. We think that, if PHC is expected to fulfill the roles described above, including further developing integrated care in the community, 25% to 30% of total health expenditure should go to primary care services in the near future. When countries decide to shift from a hospital-centric system towards a PHC-based health system, we propose that there should be an international mechanism to pay for ‘transition costs,’ because the economic gain will only appear after some years. Hence, for 3–4 years, there will be a need for ‘double financing’: keeping the hospital capacity in place while investing in PHC. It is worthwhile to explore how international agencies like the European Commission, World Health Organization, and World Bank could contribute to financing such ‘transition costs.’^14^

 Finally, we hope our propositions can inspire stakeholders, policymakers and decision-makers to see COVID-19 as an opportunity to learn to better implement change processes oriented at inclusive, equitable and person-centered integrated care.

## Ethical issues

 Not applicable.

## Competing interests

 Authors declare that they have no competing interests.

## Authors’ contributions

 JDM was asked by the editor to write a Commentary. JDM and AG discussed the analysis of the article (ref. 1). AG searched for the relevant litterature and JDM and AG selected and documented the health system challenges at micro-, meso- and macro-level. Both authors approved the final version.
